# The Need for Ethnoracial Equity in Artificial Intelligence for Diabetes Management: Review and Recommendations

**DOI:** 10.2196/22320

**Published:** 2021-02-10

**Authors:** Quynh Pham, Anissa Gamble, Jason Hearn, Joseph A Cafazzo

**Affiliations:** 1 Centre for Global eHealth Innovation Techna Institute University Health Network Toronto, ON Canada; 2 Institute of Health Policy, Management and Evaluation Dalla Lana School of Public Health University of Toronto Toronto, ON Canada; 3 Faculty of Medicine Memorial University of Newfoundland St. John's, NL Canada; 4 Institute of Biomedical Engineering University of Toronto Toronto, ON Canada

**Keywords:** diabetes, artificial intelligence, digital health, ethnoracial equity, ethnicity, race

## Abstract

There is clear evidence to suggest that diabetes does not affect all populations equally. Among adults living with diabetes, those from ethnoracial minority communities—foreign-born, immigrant, refugee, and culturally marginalized—are at increased risk of poor health outcomes. Artificial intelligence (AI) is actively being researched as a means of improving diabetes management and care; however, several factors may predispose AI to ethnoracial bias. To better understand whether diabetes AI interventions are being designed in an ethnoracially equitable manner, we conducted a secondary analysis of 141 articles included in a 2018 review by Contreras and Vehi entitled “*Artificial Intelligence for Diabetes Management and Decision Support: Literature Review*.” Two members of our research team independently reviewed each article and selected those reporting ethnoracial data for further analysis. Only 10 articles (7.1%) were ultimately selected for secondary analysis in our case study. Of the 131 excluded articles, 118 (90.1%) failed to mention participants’ ethnic or racial backgrounds. The included articles reported ethnoracial data under various categories, including race (n=6), ethnicity (n=2), race/ethnicity (n=3), and percentage of Caucasian participants (n=1). Among articles specifically reporting race, the average distribution was 69.5% White, 17.1% Black, and 3.7% Asian. Only 2 articles reported inclusion of Native American participants. Given the clear ethnic and racial differences in diabetes biomarkers, prevalence, and outcomes, the inclusion of ethnoracial training data is likely to improve the accuracy of predictive models. Such considerations are imperative in AI-based tools, which are predisposed to negative biases due to their black-box nature and proneness to distributional shift. Based on our findings, we propose a short questionnaire to assess ethnoracial equity in research describing AI-based diabetes interventions. At this unprecedented time in history, AI can either mitigate or exacerbate disparities in health care. Future accounts of the infancy of diabetes AI must reflect our early and decisive action to confront ethnoracial inequities before they are coded into our systems and perpetuate the very biases we aim to eliminate. If we take deliberate and meaningful steps now toward training our algorithms to be ethnoracially inclusive, we can architect innovations in diabetes care that are bound by the diverse fabric of our society.

## Introduction

There is clear evidence to suggest that diabetes does not affect all populations equally [1]. Among adults living with diabetes, those from ethnoracial minority communities—foreign-born, immigrant, refugee, and culturally marginalized [2]—are at increased risk of poor health outcomes [3-6]. Numerous studies have reported ethnoracial differences in glycemic control [7,8], diabetes prevalence [9], risk of diabetes complications [10], and diabetes-related mortality [11]. Data from the Centers for Disease Control indicate that non-Hispanic Black people are 2.3 times more likely to die from diabetes than their non-Hispanic White counterparts [12]. Similarly, young people living with diabetes from Black or Hispanic backgrounds are at increased risk of poor long-term glycemic control when compared to White youth [13]. The social determinants of health describe the social, economic, and physical conditions in which people are “born, live, learn, work, play, worship, and age,” as well as the impact that such environments have on health outcomes [14]. As a result of the well-accepted contribution of the social determinants toward diabetes outcomes [15], we know that ethnoracial minority populations are also more likely to experience socioeconomic adversity and subsequent challenges with diabetes management and access to care [16]. This association likely follows from the increased prevalence of various diabetes risk factors (eg, low birth weight, physical inactivity, obesity, smoking) in individuals of low socioeconomic status (SES) [16-18]. In Canada, where 21% of the population are foreign-born and live in the nation’s largest urban centers [2], people with a household income less than Can $15,000 (US $11,745) are 4 times more likely than those with a household income greater than Can $80,000 (US $62,635) to be diagnosed with type 2 diabetes (T2D) [19]. For people living with type 1 diabetes (T1D), low SES has been associated with an increased risk of poor glycemic control [20], as well as higher levels of mortality and morbidity [21].

Innovative technologies are actively being researched and developed to mitigate the burden of diabetes on patients and the health care system. Among the potential solutions, artificial intelligence (AI) appears to be well suited for diabetes management given that this chronic condition has long been guided by quantitative data collected by patients, their devices, and their care providers [22]. These data can be computationally complex for patients to make sense of on their own to inform their diabetes management [23]. AI, or the ability for “computers to think like humans” [24], has revolutionized many consumer technologies (eg, facial recognition, fraud detection, self-driving vehicles) and is now gaining momentum in the health care field. AI technologies are being developed for various areas of medicine such as medical imaging analysis [25-27], prognostication [28-30], and clinical decision support [31-33]. In diabetes care, AI is being applied for blood glucose prediction and control [34,35], identification of adverse events [36,37], lifestyle support [38,39], and predicting diabetes risk [40,41].

Despite the potential applications of AI in diabetes care, several factors may predispose these technologies to ethnoracial bias. The effectiveness of an AI algorithm is largely dependent on the quality of training data, as well as how accurately training data represent the population that will ultimately be affected by the algorithm [42]. As health data have traditionally been collected on predominantly White populations [43] or have simply omitted relevant ethnoracial information [44], algorithms trained on such data are at risk of ignoring race and ethnicity. Such ethnoracial disparities have long been present in clinical decision support tools, with various algorithms being arbitrarily corrected for race with little or no scientific justification [45]. These algorithms are widely used to inform important clinical actions such as specialist referrals [46,47] and assess candidacy for particular interventions [48,49]. In AI, where the effects of biases may be dramatic and difficult to identify [50], careless incorporation of ethnoracial data may perpetuate health inequities for those communities in most need. The alarming potential for clinical decision support tools to be algorithmically biased in favor of advantaged populations demands careful evaluation to promote their ethnoracial inclusivity. We believe that to optimize equitability, AI research should (1) establish a training population that is representative of the general population, (2) report the ethnoracial distribution of the training set, and (3) discuss potential ethnoracial limitations of the training data. To our knowledge, these simple tenets are not being met in existing diabetes AI research.

As a digital health research group preparing to build AI-based diabetes management tools [51], we want to address the challenges of promoting equity in AI and derive recommendations that can inform our work and the field at large. In an effort to better understand whether diabetes AI interventions are being designed in an ethnoracially equitable manner, we conducted a rapid case study whereby we assessed articles curated in an existing literature review of AI-based diabetes management tools. Our objectives were to (1) review ethnoracial considerations reported in past articles on AI-based diabetes support tools and (2) propose a strategy to promote ethnoracial equity in such tools in the future. This viewpoint serves to document the findings from our case study and the recommendations proposed by our group to advance ethnoracial equity in diabetes AI.

## Case Study

### Methods

We conducted a secondary analysis of 141 articles included in a high-impact literature review published in the Journal of Medical Internet Research in 2018 by Contreras and Vehi entitled “*Artificial Intelligence for Diabetes Management and Decision Support: Literature Review*” [52]. The selected review included articles describing AI technologies for diabetes management and decision support, as well as their associated challenges. We chose this review over comparable syntheses of the literature based on the short time since publication, the breadth of diabetes AI interventions included for review, and the impact that the review has had on informing the diabetes AI field.

Two members of our research team independently reviewed each of the 141 articles and selected those reporting ethnoracial data for further analysis. Articles were selected for analysis if they included an explicit description of participants’ ethnic background, racial background, or both. Articles were excluded if they were review papers, selected participants from a single ethnoracial group, or were inaccessible by the research team. The following criteria were charted for each of the selected studies: article type, diabetes type, ethnicity distribution, race distribution, number of participants, and source of data (ie, electronic medical record, electronic health record).

### Results

In screening the 141 articles included in the Contreras and Vehi review, only 10 (7.1%) were ultimately selected for secondary analysis in our case study [53-62]. Of the 131 excluded articles, 118 (90.1%) failed to mention participants’ ethnic or racial backgrounds. The remaining articles were excluded because they were review papers (n=5), selected participants from a single ethnoracial group (n=3), or were inaccessible by our research team (n=5).

The 10 articles selected for detailed analysis are summarized in Multimedia Appendices 1-3. Most articles were T2D-focused (n=8), with the remaining articles focused on T1D (n=1) and gestational diabetes (n=1). The main report types were retrospective analyses of data pulled from electronic medical records (n=5) or generated through randomized control trials (n=2). The reviewed articles reported ethnoracial data under various categories, including *race* (n=6), *ethnicity* (n=2), *race/ethnicity* (n=3), and *percentage of Caucasian participants* (n=1). Race was typically distributed between White (or Caucasian), Black (or African American), Asian, American Indian, and Alaska Native. Ethnicity was generally reported as Hispanic and non-Hispanic. Among articles specifically reporting race, the average distribution was 69.5% White, 17.1% Black, and 3.7% Asian (Multimedia Appendix 1). The 2 articles that specifically included ethnicity reported 7.2% and 21.3% Hispanic patients (Multimedia Appendix 2) [53,61]. The average distribution in articles that merged race and ethnicity was 55.4% non-Hispanic White, 8.1% non-Hispanic Black, 19.9% Hispanic, and 8.3% Asian (Multimedia Appendix 3). Only 2 articles reported inclusion of Native American participants [59,61]. The sole non-American study was performed in the Netherlands and included 97.7% Caucasian participants [60].

Several of the selected studies also included specific discussion of ethnoracial themes. Rohan et al stated that their research was limited by the homogeneity of their study population and that the generalizability of their findings should be further investigated [54]. Two more studies acknowledged that their study populations were mainly White [58,60], with one stating that their predominantly White and female demographic was “not uncommon in behavioral weight loss studies” [58]. Valdez et al intentionally oversampled racial and ethnic minorities and identified very few ethnoracial differences in health information communication patterns [61]. McCoy et al noted that race/ethnicity did not contribute to their predictions of glycemic trajectory and proposed that ethnoracial disparities in glycemic control may reflect differences in access to health care and medications [57].

## Discussion

### Ethnoracial Inequities in Diabetes AI

Diabetes AI programs are intended to improve diabetes-related health outcomes, experience, and expenditure [63,64]. However, it is unclear whether such systems benefit all populations equally. In our informal case study of 141 articles related to AI-based diabetes tools, we identified only 10 articles that specifically reported the ethnic or racial distribution of their studied patient population. We believe that this paucity of ethnoracial data in the reviewed articles significantly limits the effectiveness of the associated AI technologies. Several examples of such ethnoracial bias in clinical algorithms have been previously reported in the literature [42,45]. The long-used Framingham risk factors, which were modelled using a largely non-Hispanic White population, have recently been shown to inadequately capture risk in certain minority groups [65]. The STONE score to predict the likelihood of kidney stones in patients with flank pain equates Black race with lower risk [66]; however, an external validation study found no significant association between non-Black race and increased risk of developing kidney stones [67]. The Vaginal Birth after Cesarean (VBAC) algorithm predicts a lower likelihood of successful vaginal delivery in African American and Hispanic mothers having previously undergone cesarean section [68], while ignoring other factors (eg, private insurance status, marital status) that have been significantly associated with VBAC success [49]. A recent AI-based tool for classifying images of skin cancer was reported to perform similarly to trained experts [69]; however, the training images were predominantly of light-skinned individuals, and performance was not assessed on those with darker skin [70]. These examples highlight the importance of effective ethnoracial considerations in the development of clinical decision support tools.

Despite the promise of AI, several factors predispose AI algorithms to negative biases. One limitation of AI models is the so-called *distributional shift*, where erroneous predictions result from a mismatch between the training population and the population on which the model is used. Such a mismatch can result from “bias in the training set, change over time, or use of the system in a different population” [50]. Essentially, the robustness of AI algorithms is dependent upon the degree to which the training population represents the target population [71]. In addition to the distributional shift phenomenon, the complexity and black-box nature of AI algorithms often obfuscates underlying errors or biases, specifically when compared to simpler rule-based systems [50]. The detection of such biases in AI algorithms often requires careful consideration of model behavior in response to changing inputs [72]. In the case of ethnoracial data, the omission of such information could result in a distributional shift based on ethnicity, race, or both in resultant models, which may be difficult for researchers to identify at the time of development.

Given the clear ethnic and racial differences in diabetes biomarkers, prevalence, and outcomes [7-10,12,73], the inclusion of ethnoracial data is also likely to improve the accuracy of predictive models. The predictive value of race and ethnicity is well-documented in the literature, where they have been shown to independently predict health decline for adults living with diabetes [74,75]. The impact of specific risk factors for T2D have even been shown to vary for both sex and race, with the most predictive factors being waist circumference in Black men, 2-hour glucose from an oral glucose tolerance test in Black women, and fasting glucose in both White men and White women [76]. As a result of these associations between diabetes outcomes and ethnoracial information, the consideration of ethnoracial data is likely to enhance both the accuracy and generalizability of resultant AI-based diabetes tools.

In those articles we reviewed that did include ethnoracial information, there was very little standardization in terms of how these data were reported (eg, *race*, *race and ethnicity*, *race/ethnicity*). Race distinguishes individuals based on ancestry and combinations of physical characteristics, whereas ethnicity focuses on behavior and culture in addition to physical features [77]. Inconsistent reporting of ethnic and racial information hinders the ability to perform meta-analyses across multiple data sets and may limit ethnoracial equity in future AI applications [78]. In their writings on eliminating health disparities, Fremont and Lurie state that data pertaining to race and ethnicity are collected by a variety of sources, but “the utility of these data is constrained by ongoing problems with reliability, completeness, and lack of comparability across data sources” [79]. Though differences in the reporting of ethnoracial data are expected across jurisdictions, we propose that authors attempt to report such data in a manner that is easily comparable to locally available data. For example, the US census reports race and ethnicity separately, with ethnicity being used to determine whether an individual is of “Hispanic origin or not” and race being categorized as “White, Black or African American, Asian, American Indian or Alaska Native, Native Hawaiian or Other Pacific Islander, and other” [80]. Kiran et al recently assessed Canadian patient perspectives on routinely being asked about their race and ethnicity through a sociodemographic questionnaire [81]. They found that patients were not uncomfortable disclosing their race and ethnicity and intuitively understood how the data could be helpful for their health care providers. Their work has subsequently informed the collection of race-based data during the COVID-19 pandemic [82]. These are just two examples of standards that will allow for comparison with locally available data and in turn enable the assessment of ethnoracial generalizability and cultural competence in diabetes AI algorithms.

In considering the average race distribution of the reviewed studies, the proportions for White (69.5%) and Asian race (3.7%) were slightly lower than those values reported in recent US census data (76.3% and 5.9%, respectively). The opposite was true for the Black race, which accounted for 17.1% of study participants but only 13.4% of US census participants. In those studies reporting race and ethnicity as a combined variable, the average proportion of non-Hispanic whites (55.4%) was slightly lower than the census value of 60.1% [83]. These findings likely follow from the high prevalence of diabetes in the non-Hispanic Black population, specifically when compared to the non-Hispanic White and Asian populations [84]. One particularly worrisome finding was that data from Native American participants were reported in just 2 studies [59,61], despite making up an estimated 1.3% of the American population [83] and being the ethnoracial group with the highest age-adjusted prevalence of diagnosed diabetes [9]. Poor Indigenous representation in health and governmental data sets has been previously reported in the literature [85,86]. In Canada, where Indigenous peoples account for 4.9% of the population [87] and are disproportionately affected by diabetes [88], failure to include Indigenous data when training diabetes AI models could propagate existing issues of health inequity and structural racism in this population [89,90].

### A Simple Screening Tool to Assess Ethnoracial Equity in Diabetes AI

Detailed guidelines currently exist for the development of trustworthy and human-centric AI technologies [91]. However, we believe there is a need for simple tools to screen the ethnic and racial generalizability of AI in health care. Based on the findings from our case study, we have developed a short screening tool that researchers and clinicians may use to assess ethnoracial equity in research describing AI-based diabetes interventions. The rationale and structure of this tool borrows from the Jadad scale [92], which was conceived by the founder of our research group over two decades ago and is widely used to assess the methodological quality of a clinical trial [93,94]. We propose the following set of five questions to consider the ethnoracial relevance of diabetes AI:

1. Did the research explicitly describe the disease under study (eg, T1D, T2D, both)? (1a) Did the research describe ethnoracial differences in disease prevalence, biomarkers, and outcomes?
2. Did the research clearly describe the sources of data used in the training data set (eg, electronic medical record, administrative data repository, research registry)? (2a) Did the research describe ethnoracial limitations in the sources of data?
3. Did the research explicitly report the ethnic and racial backgrounds of individuals in the training data set? (3a) Are ethnic and racial backgrounds reported in a manner that is easily comparable to local census data?
4. Do the ethnic and racial distributions in the training data set accurately represent the population on which the algorithm will be used? (4a) Did the research articulate limitations of the ethnic and racial distributions in the training data set?
5. Did the research describe strategies to mitigate ethnoracial bias in the algorithm?

Although we feel that our proposed tool will be helpful in assessing clinical AI algorithms generally, it will be particularly important in the development of diabetes AI. We believe that these innovations will fail to serve the diabetes community if they are not trained on ethnoracially diverse data. As AI-based systems become integrated into important aspects of diabetes management, such ethnoracial inequities in model development could ultimately be dangerous for minority groups whose biomarkers and outcomes may differ from the general population. In the Contreras and Vehi review, most studies focused on T2D self-management, clinical decision support, and prediction tools. Each of these dimensions of diabetes care can be affected by ethnoracial factors. For example, adherence to T2D medications to achieve euglycemia is demonstrably driven by cultural beliefs, values, social factors, religion, health literacy, and language barriers [95,96]. Similar issues are likely to follow in the T1D space, where AI algorithms are currently focused on automated insulin delivery systems but will likely shift toward the above dimensions in the near future [63,97,98].

Addressing ethnoracial bias in diabetes AI has been made even more critical by the coronavirus disease 2019 (COVID-19) pandemic [99]. There is growing evidence to support a “bidirectional relationship between COVID-19 and diabetes” [100]. Research suggests that diabetes is a risk factor for rapid progression and poor prognosis of COVID-19 [101,102]. New-onset diabetes is also being reported in previously healthy individuals diagnosed with COVID-19 [103-105], which may reflect coronavirus-inflicted damage to insulin-producing cells [106,107]. We are concerned by these findings from a health equity lens, given that COVID-19 has been found to disproportionately affect ethnoracial minorities. The Centers for Disease Control and Prevention have already determined that individuals from Black and American Indian or Alaska Native communities have a rate of hospitalization or death from COVID-19 that is 5 times greater than that of their White counterparts [108]. It stands to reason that the increased prevalence of both COVID-19 and diabetes in ethnoracial minority groups and the relationship between these two conditions require ethnoracial considerations in all aspects of diabetes care.

At this unprecedented time in history, AI can either mitigate or exacerbate disparities in health care. Future accounts of the infancy of diabetes AI must reflect our early and decisive action to confront ethnoracial inequities before they are coded into our systems and perpetuate the very biases we aim to eliminate [45]. If we take deliberate and meaningful steps now toward training our algorithms to be ethnoracially inclusive, we can architect innovations in diabetes care that are bound by the diverse fabric of our society.

## Appendix

Multimedia Appendix 1 Distribution of articles specifically reporting race.

Multimedia Appendix 2 Distribution of articles specifically reporting ethnicity.

Multimedia Appendix 3 Distribution for articles reporting race and ethnicity as a merged variable.

## Abbreviations

AI: artificial intelligence
SES: socioeconomic status
T1D: type 1 diabetes
T2D: type 2 diabetes
VBAC: Vaginal Birth after Cesarean
