# Supplementary material for: The Need for Ethnoracial Equity in Artificial Intelligence for Diabetes Management: Review and Recommendations
Source: J Med Internet Res. 2021 Feb 10;23(2):e22320. doi: 10.2196/22320 (PMC7904401; doi:10.2196/22320)
Supplement: Multimedia Appendix 1 [file jmir_v23i2e22320_app1.docx]

**Multimedia Appendix 1.**Distribution of articles specifically reporting race

| **Lead author, Year** | **White** | **Black** | **Asian** | **Native American** ^a^ | **Other** |
| --- | --- | --- | --- | --- | --- |
| Nunes, 2016[^53^](https://paperpile.com/c/Mk3QOF/nFto) | 77.6% | 9.6% | 3.1% | 0% | 9.5% |
| Anderson, 2015[^55^](https://paperpile.com/c/Mk3QOF/v5OL) | 61.0% | 14.6% | 2.1% | 0% | 22.3% |
| DuBrava, 2017[^56^](https://paperpile.com/c/Mk3QOF/o5RQ) | 76.0% | 14.7% | 1.1% | 0% | 8.2% |
| Everett, 2018[^58^](https://paperpile.com/c/Mk3QOF/qmGn) | 70.9% | 25.5% | 0% | 0% | 3.6% |
| Valdez, 2017[^61^](https://paperpile.com/c/Mk3QOF/0BFF) | 61.8% | 21.3% | 12.3% | 1.1%^a^ | 3.5% |
| Average | 69.5% | 17.1% | 3.7% | 0.2% | 9.4% |

^a^ Reported as American Indian/Alaska Native
