# Supplementary material for: The Need for Ethnoracial Equity in Artificial Intelligence for Diabetes Management: Review and Recommendations
Source: J Med Internet Res. 2021 Feb 10;23(2):e22320. doi: 10.2196/22320 (PMC7904401; doi:10.2196/22320)
Supplement: Multimedia Appendix 2 [file jmir_v23i2e22320_app2.docx]

**Multimedia Appendix 2.**Distribution of articles specifically reporting ethnicity

| **Lead author, Year** | **Hispanic** | **Non-Hispanic** | **Other** |
| --- | --- | --- | --- |
| Nunes, 2016[^53^](https://paperpile.com/c/Mk3QOF/nFto) | 7.2% | 80.3% | 12.2% |
| Valdez, 2017[^61^](https://paperpile.com/c/Mk3QOF/0BFF) | 21.3% | 78.7% | 0% |
| Average | 14.3% | 79.5% | 6.1% |
