# Supplementary material for: The Need for Ethnoracial Equity in Artificial Intelligence for Diabetes Management: Review and Recommendations
Source: J Med Internet Res. 2021 Feb 10;23(2):e22320. doi: 10.2196/22320 (PMC7904401; doi:10.2196/22320)
Supplement: Multimedia Appendix 3 [file jmir_v23i2e22320_app3.docx]

**Multimedia Appendix 3.**

Distribution for articles reporting race and ethnicity as a merged variable

| **Lead author, Year** | **NHW**^a^ | **NHB**^a^ | **Hispanic** | **Asian**^b^ | **Native American** | **Other** |
| --- | --- | --- | --- | --- | --- | --- |
| Rohan, 2011[^54^](https://paperpile.com/c/Mk3QOF/aH2z) | 74.5% | 4.6% | 11.7% | 0% | 0% | 9.2% |
| McCoy, 2017[^57^](https://paperpile.com/c/Mk3QOF/OTkl) | 57.6% | 14.7% | 11.0% | 7.5% | 0% | 9.3% |
| Hazlehurst, 2014[^59^](https://paperpile.com/c/Mk3QOF/HqTd) | 34.0% | 5.0% | 37.0% | 17.5%^b^ | 1.0% | 5.5% |
| Average | 55.4% | 8.1% | 19.9% | 8.3% | 0.3% | 8.0% |

^a^ NHW: non-Hispanic White; NHB: non-Hispanic Black

^b^ Reported as Asian/Pacific Islander
